# Anti-Diabetic Potential of *Ocimum gratissimum* Leaf Fractions in Fortified Diet-Fed Streptozotocin Treated Rat Model of Type-2 Diabetes

**DOI:** 10.3390/medicines4040073

**Published:** 2017-10-11

**Authors:** Stanley I. R. Okoduwa, Isamila A. Umar, Dorcas B. James, Hajiya M. Inuwa

**Affiliations:** 1 Department of Biochemistry, Ahmadu Bello University, Zaria 811104, Nigeria; iaumar2003@yahoo.co.uk (I.A.U.); dbjams16187@yahoo.com (D.B.J.); inuwahm@yahoo.com (H.M.I.); 2 Directorate of Research and Development, Nigerian Institute of Leather and science Technology, Zaria 810221, Nigeria

**Keywords:** Type-2 diabetes, *Ocimum gratissimum*, hyperglycaemia, hyperlipidaemia, rats

## Abstract

**Background**: *Ocimum gratissimum* (OG) is used in the traditional management of diabetes in Nigeria. This study investigated the anti-diabetic potential of OG leaf fractions (OGLF) in a rat model of Type-2 diabetes (T2D). **Methods**: Methanol crude extract of OG leaf was fractionated with solvents of increasing order of polarity (*n*-hexane, chloroform, ethyl-acetate, *n*-butanol and water). The anti-diabetic potential of the fractions was evaluated in vivo. T2D was induced in Albino Wistar rats and treated with OGLF. **Results**: The T2D rats showed significant elevation in serum levels of fasting blood glucose (FBG), liver and kidney function biomarkers. At 4-week of intervention with OGLF, the untreated diabetic control group maintained severe hyperglycaemia in the presence of 61.7% serum insulin, 17.3% pancreatic β-cell function (HOMA-β) and 51.5% Insulin sensitivity. The glucose tolerance ability was enhanced in the *n*-butanol-fraction (OGb) treated group. With 74.8% available serum insulin and 38.6% improvement in insulin sensitivity, the OGb treated group had a 63.5% reduction in FBG and it was found to be most effective as it ameliorates a majority of the changes caused in the studied parameters in diabetic rats. **Conclusions**: The data from this study suggest that OGb fraction is a potential candidate for the development of an effective drug for the management of T2D.

## 1. Introduction

Diabetes mellitus is an important chronic metabolic disorder of public health concern. It occurs either as a result of pancreatic defects in insulin secretion or the failure of the receptor cells to effectively utilise secreted insulin or both [1,2,3]. Hyperglycaemia is a common consequence of uncontrolled diabetes, which may over time lead to serious damage to vascular tissue, heart, eyes, nerves and kidneys [1,3,4]. Diabetes is no longer a disease of predominantly rich nations; its prevalence is steadily increasing across the globe—age [3,5], gender, socioeconomic status and ethnicity of the individual [6] notwithstanding. Globally, an estimated 422 million adults were reported to be living with diabetes in 2014, compared to 108 million in 1980; a dramatic rise in prevalence rate from 4.7% to 8.5% in the adult population [3]. In 2015 in Nigeria alone, 1.56 million cases were reported including 105,091 deaths [2]. Every six seconds a person dies from diabetes in the world. In 2012 there were 1.5 million deaths worldwide directly caused by diabetes and about 95% of all cases reported were attributed to Type-2 diabetes mellitus (T2DM), commonly called Type-2 diabetes (T2D) [2,3].

The ever-increasing incidence of T2D has become a severe threat to humanity. In spite of advancements recorded to date on basic and clinical investigations into diabetes (especially in Nigeria and environs where healthcare resources are limited) a properly effective final remedy does not exist. The existing therapeutic remedies (which include just four core categories of oral hypoglycemic agents viz: biguanide, glucosidase inhibitors, sulfonylurea and pioglitazone) are grossly inadequate [7]. More so, they are synthetic drugs that have several strong adverse effects like liver problems, lactic acidosis and diarrhoea [4,7,8]. Researchers have thus turned their collective attention to nature, and the vast diversity of global flora for less toxic but viable solutions.

Plants with known and suspected therapeutic potencies have long been used in alternative and complementary medicine. Numerous scientific reports exist, describing the relatively low toxicity and effectiveness of select plants in the management of diabetes [4,9,10]. In Nigeria, *Ocimum gratissimum* is one of the many plants found to lower glycaemia in Type-1 diabetes (T1D) [8,11,12].

*Ocimum gratissimum* (OG) is an aromatic medicinal plant which belongs to the Lamiaceae family. It is popularly known as scent leaf. It is used in cooking due to its minty aromatic flavour. In Nigeria, the plant is called “Effinrin-na” by the Yoruba speaking tribe, “Alumokho” in Esan, “Nchanwu” in Igbo, “Aramogbo” in Edo, and in the northern part of Nigeria—the Hausas call it “Daidoya” [13]. Traditionally, OG has been used for the treatment of headache, diarrhoea, warts, worms, kidney infections and diabetes mellitus [13,14].

In a recent review, it was reported that alloxan and/or streptozotocin (STZ) were the most frequently used diabetogenic agents globally [8,15]. High doses of these chemicals are cytotoxic to the pancreatic β-cells, giving rise to insulin deficiency, similar to Type-1 diabetes (T1D) with resultant hyperglycaemia [15,16]. Interestingly, there is a scarcity of literature on the antidiabetic potential of OG leaf fraction on T2D.

Even though several reports attest to the potency of OG leaf in the management of diabetes, it is unfortunate that a large number of those studies were conducted using either normoglycaemia or animal models of T1D [11,12]. Induction of diabetes using either >100 mg/kg body weight (bw) of Alloxan or >40 mg/kg of streptozotocin, results in insulin deficiency typical for T1D. These were the methods used predominantly by most previous investigators such as Okon (2012) in Calabar [17], Asuquo et al. (2009) in Calabar [18], Onaolapo et al. (2012), Onaolapo and Onaolapo (2012) in Osun State [19,20], Mohammed et al. (2007) in Zaria [21], Egesie et al. (2006) in Jos [12], Igoli et al. (2005) in Calabar [14] and Aguiyi et al. (2000) [22]. Sadly, screening of medicinal plants intended for management of T2D may not yield positive results when inappropriate animal models are used.

Type-2 diabetes mellitus is no doubt a major dilemma in the world today, especially in the way it deteriorates the quality of human life. Thus far, due in part to lack of a suitable T2D model, most anti-diabetic research has been on T1D [11,15]. A typical procedure towards development of reliable therapeutic strategy for the likely cure of a disease is the study of the pathophysiology of the disease in an appropriate animal model [23,24]. This study therefore seeks to investigate the anti-diabetic (anti-hyperglycaemic, anti-hyperlipidaemic, pancreatic, nephroprotective and hepatoprotective) potential of OG leaf fractions (OGLF) in a unique rat model of T2D otherwise known as the fortified diet-fed streptozotocin-treated (FDF-STZ) rat model of T2D. The model has been characterized with insulin resistance and development of hyperglycaemia in the presence of significant high level of insulin. Also, it was distinguished for being sensitive to T2D drugs like metformin, pioglitazone and glibenclamide [23]. This study is the first of its kind to utilize the new model of T2D for pharmacological screening.

## 2. Materials and Methods

### 2.1. Plant Materials

Leaves of *Ocimum gratissimum* (OG) were harvested from local farm in Samaru, Zaria, Kaduna state, Nigeria in May 2015. They were identified, authenticated and assigned the voucher specimen number 1285 at the Herbarium Unit, Department of Biological Sciences, Ahmadu Bello University, Zaria Nigeria. They were air-dried, pulverised and stored in air-tight containers for further analysis.

### 2.2. Experimental Animals

Wistar Albino rats, weighing 150–200 g, were used for the research. They were purchased from the animal house of the Department of Pharmacology, Ahmadu Bello University, Zaria Nigeria. The rats were kept in well aerated cages where bedding was replaced each day, at a room temperature of about 27 °C and 12 h light/dark cycle. They were allowed to acclimatize for two weeks prior to experimentation. During this period, they were all provided with the same commercially available rat pellets and tap water *ad libitum.* The Institutional Animal Research Ethics Committee reviewed and certified the experimental protocol (AREC/EA15/055) in conformity with guidelines that are in compliance with National and International Laws and Guidelines for Care and Use of Laboratory Animals in Biomedical Research. Strict adherence to the Ethical Committee’s directives was observed. Efforts were made to reduce suffering by the animals. The criterion of anaesthesia was the lack of body or limb movement in reaction to a standardised tail clamping stimulus.

### 2.3. Chemical and Reagents

Strepozotocin (STZ) was procured from Adooq Bioscience, LLC, Irvine, CA, USA, fructose (Kem Light Laboratories PVT Ltd, Mumbai, India), Mission Cholesterol Meter (ACON Lab. Inc., San Diego, CA, USA), Simas Margarine (PT Salim Ivomas Pratama Tbk, Jakarta, Indonesia), On-call plus glucometer, Normal diet feed (Grand Cereals Limited, Jos, Nigeria); Liver enzyme kits (Biovision, Milpitas, CA, USA), rat insulin ELISA kit (Mercodia AB, Uppsala, Uppsala, Sweden), rat C-peptide ELISA kit (WKEA Med supplies, Changchun, China) and all other reagents were of existing analytical grade and procured from appropriate manufacturing companies.

### 2.4. Extraction of the Plants’ Crude Extracts

One thousand two hundred and fifty grams of the powdered samples were extracted with 80% methanol at room temperature as described previously [25]. The extracts were evaporated to yield the crude residue.

### 2.5. Fractionation of the Plants’ Crude Extracts

One hundred grams of the crude extract was dissolved in distilled water and partitioned by liquid-liquid fractionation using organic solvents in an increasing order of polarity (*n*-hexane, chloroform, ethyl-acetate, *n*-butanol and water). In brief, the crude extract was first soaked in *n*-hexane in a separating funnel, shaken and allowed to stand for phase separation into two fractions. The *n*-hexane portion was cautiously decanted after separation and extra *n*-hexane solvent was added and the procedure repeated until no more colour change was observed in the solvent addition. The *n*-hexane soluble portion was obtained and allowed to dry at room temperature to obtain the *n*-hexane fraction. The same process was repeated for chloroform, ethyl-acetate and *n*-butanol to obtain the appropriate solvent fractions. The resultant insoluble residue was dissolved in water and labelled as aqueous fraction. Each fraction obtained was concentrated using a rotary evaporator, the residual solvent in the extract was allowed to evaporate at room temperature to an invariable weight. Each of the fractions obtained were screened in vivo for anti-diabetic potential using FDF-STZ T2D rat model.

### 2.6. Phytochemical Analysis of the Fractions

The presence or absence of some specific phytochemicals was examined in the OG leaf fractions using standard phytochemical screening procedures described by Trease and Evans [26]. 

### 2.7. Oral Acute Toxicity Studies

The median lethal dose (LD_50_) of the extracts in Wistar Albino rats was determined using method described by Lorke [27]. The procedure for determining the LD_50_ involves the increment of the concentration of each extract administered to the rats (per kg bodyweight) in each group consisting of six (6) rats per group. The concentrations used ranged from 10 mg/kg up to 5000 mg/kg respectively. The animals were monitored constantly for changes in respiration, palpitations, behaviour, toxicity, and other signs of morbidity and/or mortality starting at 24 h for up to 7 days.

### 2.8. Determination of Effective Minimum Therapeutic Dose

The hypoglycaemic activity of OG leaf extract was evaluated in order to determine the minimum dose of extract to be administered. Diabetic rats were divided into several groups (G_100_, G_150_, G_200_, ... and DC) comprising of three rats each. The animals in group DC served as diabetic untreated control whereas the other groups (G_100_, G_150_, G_200_, ...) were administered with the methanol crude extract of OG at a single dose of 100, 150, 200 mg/kg etc. respectively. Percentage change in blood glucose was estimated at 0, 1, 2, 3 and 4 h calculated for each group using the following formula:

$$\%\;\mathrm{Variation}\;\mathrm{in}\;\mathrm{glycaemia}\;=\frac{\mathrm{Go}\;-\;\mathrm{Gt}}{\mathrm{Go}}\;\times \;100.$$

where Go and Gt were the values of initial blood glucose (0 h) and blood glucose at time 1, 2, 3 and 4 h respectively. The blood glucose levels at different time intervals of different groups were compared. The extract dose that lowered the glucose level by 25% at 4 h was considered the minimum hypoglycaemic dosage [28].

### 2.9. Induction of Type 2 Diabetes

The induction procedure for Type 2 diabetes was carried out as previously described [23]. In brief, commercially available Normal Diet Feed (NDF) was fortified with margarine in a ratio of 10 g NDF per gram of margarine. This was administered, along with 20% fructose solution as drinking water, to the rats *ad libitum* for six weeks after which they were fasted overnight and injected (*ip*) with STZ (dissolved in a citrate buffer pH 4.5) at a single low dose of 35 mg/kg bw. In the first 24 h after STZ induction, the rats were provided with 5% glucose solution as drinking water.

### 2.10. Confirmation of Diabetes

Pre-confirmation of diabetes was done three days after STZ induction, with On-call plus glucometer using blood samples obtained via tail puncture of the rats. At day 10 following the pre-confirmation, animals with fasting blood glucose (FBG) ≥300 mg/dL were confirmed diabetic and incorporated in the study as diabetic animals [29].

### 2.11. Grouping of Experimental Animals 

The rats were divided into eight groups of six rats each:

Group NC: Normal control: normoglycemia rats given distilled water.
Group DC: Diabetic control: diabetic rats treated with vehicle alone.
Group OGh: Diabetic rats treated with *Ocimum gratissimum n*-hexane fraction.
Group OGc: Diabetic rats treated with *Ocimum gratissimum* chloroform fraction.
Group OGe: Diabetic rats treated with *Ocimum gratissimum* ethyl-acetate fraction.
Group OGb: Diabetic rats treated with *Ocimum gratissimum n*-butanol fraction.
Group OGa: Diabetic rats treated with *Ocimum gratissimum* aqueous fraction.
Group PC: Positive control: diabetic rats treated with metformin (500 mg/kg bw)

### 2.12. Treatment of Animals with Plant Fractions

The plant fractions were administered at a dose of 250 mg/kg body weight by oral intubation (that is through oesophageal cannula) to the diabetic rats daily for 4 weeks. This dose was derived from the minimum oral therapeutic study described above.

### 2.13. Observation of Animals during and after Treatment

Daily feed/fluid intake and weekly body weight changes were measured during the entire experimental period.

### 2.14. Oral Glucose Tolerance Test (OGTT)

Three hours after the last dose of treatment, OGTT was carried out. Rats were orally dosed with a D-glucose solution (2.0 g/kg bw) and blood glucose levels were afterwards measured at 0 (just prior to oral glucose dosing), 30, 60, 90, and 120 min after the oral dosing of glucose using blood samples obtained from tail puncture of the rats.

### 2.15. Sample Collection

At the end of the experimental phase, all animals were fasted overnight and thereafter euthanized by halothane anaesthesia. Blood was collected into plain bottles through cardiac puncture and placed immediately on ice for 3 h, then centrifuged at 3000 rpm for 15 min to obtain serum which was separated and stored at −30 °C until further investigations. The pancreas from each rat was cut and placed in a 10% neutral buffered formalin solution and preserved at room temperature for histopathological study.

### 2.16. Biochemical Parameters

The serum insulin levels were measured by an enzyme linked immunosorbent assay (ELISA) method using an ultrasensitive rat insulin ELISA kit (Mercodia AB, Uppsala, Sweden) and Rat C-peptide ELISA kit (WKEA Med supplies, Changchun, China) in a multi plate ELISA reader (Biorad-680, BIORAD Ltd., Tokyo, Japan). The serum lipid profile was determined using Mission Cholesterol Meter (ACON Lab. Inc., San Diego, CA, USA). Serum urea, uric acid and creatinine levels, as well as liver function enzymes: aspartate and alanine transaminases (AST and ALT) and alkaline phosphatase (ALP) were measured using an Automated Chemistry Analyzer (Labmax Plenno, Labtest Co. Ltd., Lagoa Santa, Brazil) with commercial assay kits. Homeostatic model assessment (HOMA-IR and HOMA-β) scores and insulin sensitivity were calculated using fasting serum insulin and FBG concentrations measured at the end of the experimental period according to the formula of Matthew et al. [30]:

$$\text{HOMA-IR }=\;\frac{\mathrm{Insulin}\;(U/L)\times \;\mathrm{Blood}\;\mathrm{glucose}\;(\mathrm{mmol}/L)\;}{22.5}$$

$$\text{HOMA-}\beta \;=\;\frac{20\;\times \;\mathrm{Insulin}\;(U/L)\;}{\mathrm{Blood}\;\mathrm{glucose}\;(\mathrm{mmol}/L)\;}\;-\;3.5$$

$$\mathrm{Insulin}\;\mathrm{Sensitivity}\;(\mathrm{IS})\;=\;\frac{1\;}{\mathrm{Log}[\mathrm{fasting}\;\mathrm{insulin}(U/L)]\;\times \;\mathrm{Log}[\mathrm{fasting}\;\mathrm{glucose}\;(\mathrm{mmol}/L)]\;}$$

Determination of the percentages of HOMA-β cell function, insulin sensitivity, improvement in insulin sensitivity and circulating serum insulin was calculated based on the derivation from Matthew et al. [30] as follows:

$$\mathrm{Percentage}\;\beta \times \mathrm{cell}\;\mathrm{function}\;\mathrm{in}\;\mathrm{exprimental}\;=\;\frac{\mathrm{HOMA}\;\beta \;-\;\mathrm{cell}\;\mathrm{fucntion}\;\mathrm{in}\;\mathrm{Expt}.\;}{\mathrm{HOMA}\;\beta \;-\;\mathrm{cell}\;\mathrm{function}\;\mathrm{in}\;\mathrm{normal}\;\mathrm{control}\;}\;\times \;100$$

$$\mathrm{Percentage}\;\mathrm{Insulin}\;\mathrm{in}\;\mathrm{experimental}\;=\;\frac{\mathrm{Insulin}\;\mathrm{senitivity}\;\mathrm{in}\;\mathrm{Expt}.\;}{\mathrm{Insulin}\;\mathrm{Senitivity}\;\mathrm{in}\;\mathrm{normal}\;\mathrm{control}\;}\times 100$$

$$\mathrm{Percentage}\;\mathrm{Improvement}\;\mathrm{in}\;\mathrm{Insulin}\;\mathrm{Sensitivity}\;\mathrm{of}\;\mathrm{Expt}.\;=\;\frac{\left(\mathrm{Insulin}\;\mathrm{sensitivity}\;\mathrm{of}\;\mathrm{Expt}\right)-(\mathrm{Insulin}\;\mathrm{Sensitivity}\;\mathrm{of}\;\mathrm{DC})\;}{(\mathrm{Insulin}\;\mathrm{Sensitivity}\;\mathrm{of}\;\mathrm{DC})\;}\;\times \;100$$

$$\mathrm{Percentage}\;\mathrm{Circulating}\;\mathrm{Serum}\;\mathrm{Insulin}\;\mathrm{of}\;\mathrm{Expt}.\;=\;\frac{(\mathrm{Serum}\;\mathrm{Insulin}\;\mathrm{of}\;\mathrm{Expt}.)\;}{(\mathrm{Serum}\;\mathrm{Insulin}\;\mathrm{of}\;\mathrm{NC})\;}\;\times \;100$$

where “Expt.” = Experimental test group; DC = Diabetic control group (Diabetic rats without treatment); NC = Normal control (Normoglycaemic) group.

### 2.17. Histopathological Examination of Pancreatic Tissue

The formalin preserved pancreatic tissues were treated according to a standard laboratory protocol for paraffin embedding. Sections were cut at a size of 4 mm then slides were deparaffinised in *p*-xylene and rehydrated in changes of ethanol concentrations (100%, 80%, 70%, 50%) and rinsed with water. Slides were stained in haematoxylin for 5 min and rinsed with water and counterstained in eosin, mounted on slides with cover-slips and viewed under a Leica scanner at ×100 magnification.

### 2.18. Statistical Analysis

The computer software Statistical Package for the Social Sciences (SPSS Cary, NC, USA) version 20.0 was used for analysis of all statistical data. The data were analysed by analysis of variance (ANOVA) and *post hoc* tests. The results are expressed as mean ± SD. Differences between fractions and animal groups were compared using Duncan Multiple Range Test (DMRT). A value of *p* < 0.05 were considered significant.

## 3. Results

### 3.1. Percentage Yield and Phytochemical Screening of the Plant Fractions

Table 1 presents the characteristics features and percentage yield of the various fractions of the leaf. The highest yield was recorded in aqueous fraction (33.7%) when compared with the *n*-hexane, ethyl acetate, chloroform and *n*-butanol fraction.

Table 2 shows the type of phytoconstituents present in the various leaf fractions. The presence of tannin and saponin were detected in *n*-butanol and aqueous factions but absent in *n*-hexane, ethyl acetate and aqueous fractions. Alkaloid and flavonoids were not detected in aqueous and *n*-hexane fractions respectively. In all the fractions, anthraquinones was not detected.

### 3.2. Determination of Effective Minimum Oral Therapeutic Dose of OG Leaf Extracts

Table 3 reveals the effective minimum oral therapeutic dose of OG leaf extract. It was observed that OG leaf extract dose of 200 mg/kg bw decreased the blood glucose of diabetic rats by 20.9%, at 4 h whereas 250 mg/kg bw was able to decrease the blood glucose of diabetic rats by 29.3% at 4 h. The 250 mg/kg bw dose was considered as the effective minimum oral therapeutic dose for this study.

### 3.3. Mean Feed and Fluid Intake

**Figure 1:**
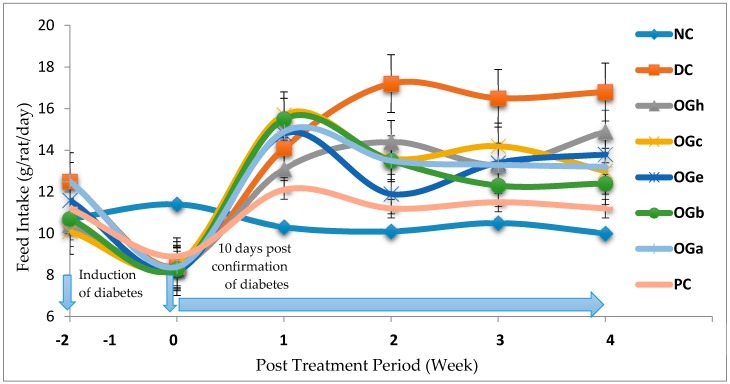
Effect of treatment with *Ocimum gratissimum* leaf fractions (250 mg/kg bw) on feed intake throughout the experimental duration. Data are presented as mean of 6 animals per group. NC: Normal control; DC: Diabetic control; PC: Positive control (Metformin (500 mg/kg bw); OGh: *Ocimum gratissimum n*-hexane fraction; OGc: *Ocimum gratissimum* chloroform fraction; OGe: *Ocimum gratissimum* ethyl-acetate fraction; OGb: *Ocimum gratissimum n*-butanol fraction; OGa: *Ocimum gratissimum* aqueous fraction.

**Figure 2:**
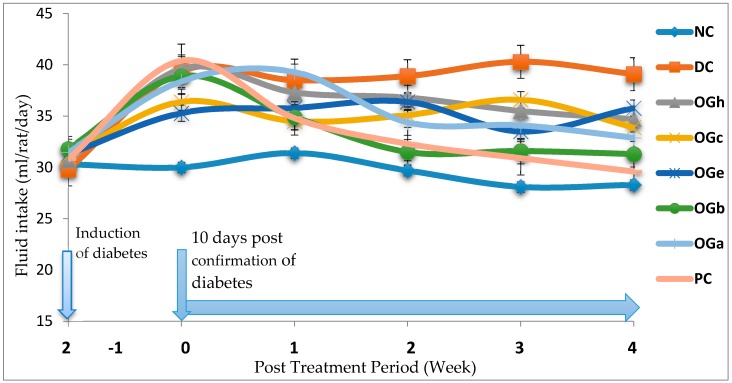
Effect of treatment with *Ocimum gratissimum* leaf fractions (250 mg/kg bw) on fluid intake throughout the experimental duration. Data are presented as mean of 6 animals per group. NC: Normal control; DC: Diabetic control; PC: Positive control (Metformin 500 mg/kg bw); OGh: *Ocimum gratissimum n*-hexane fraction; OGc: *Ocimum gratissimum* chloroform fraction; VAe: *Ocimum gratissimum* ethyl-acetate fraction; OGb: *Ocimum gratissimum n*-butanol fraction; OGa: *Ocimum gratissimum* aqueous fraction.

Figure 1 and Figure 2 reveal the trends of mean feed and fluid intake respectively for the experimental animals throughout the experimental duration. Significant decrease in feed intake was observed a few days after induction among the STZ induce groups when compared with the normal control (NC) group that were not induced. A week after confirmation of diabetes, the feed intake increased significantly among the diabetic confirmed groups. Between the second and fourth weeks of treatment with the OGLF, it was observed that the feed intake among the diabetic rats treated, decrease significantly (*p* < 0.05) when compared with the untreated diabetic group (DC). The fluid intake significantly (*p* < 0.05) increased among the diabetic groups when compared with the normal control group. Throughout the experimentation, it was observed that the fluid intake among the diabetic rats treated with OGLF was significantly (*p* < 0.05) lower than the DC but higher than the NC group. The *n*-butanol fraction and the metformin treated group (PC) had a comparable fluid intake towards the end of the fourth week (Figure 2).

### 3.4. Effect of Treatment on Total Body Weight

**Figure 3:**
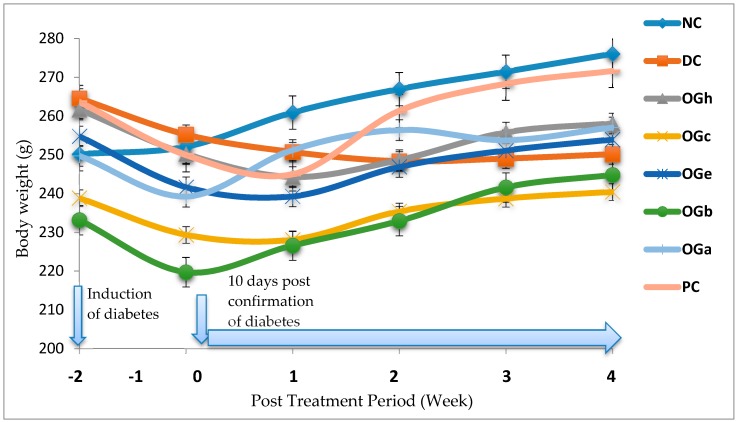
Effect of treatment with *Ocimum gratissimum* leaf fractions (250 mg/kg bw) on body weight of diabetic rats. Data are presented as mean of 6 animals per group. NC: Normal control; DC: Diabetic control; PC: Positive control (Metformin 500 mg/kg bw); OGh: *Ocimum gratissimum n*-hexane fraction; OGc: *Ocimum gratissimum* chloroform fraction; OGe: *Ocimum gratissimum* ethyl-acetate fraction; OGb: *Ocimum gratissimum n*-butanol fraction; OGa: *Ocimum gratissimum* aqueous fraction.

Figure 3 shows the changes in body weight of all the experimental animals. At day 10 post-confirmation of diabetes, a decrease in body weight was observed among the diabetic group. From the second week of treatment with the OGLF, a significant decrease (*p* < 0.05) in weight increase was observed among the diabetic rats treated with OGLF and PC groups when compared with the NC group. On the contrary, a decrease in body weight was observed in the DC group. With respect to all the diabetic treated groups, the OG *n*-butanol fraction treated group and the PC group had increased in body weight significantly (*p* < 0.05) in a comparable manner to the NC group (Figure 3).

### 3.5. Effect of Post-Treatment on Blood Glucose Levels

**Figure 4:**
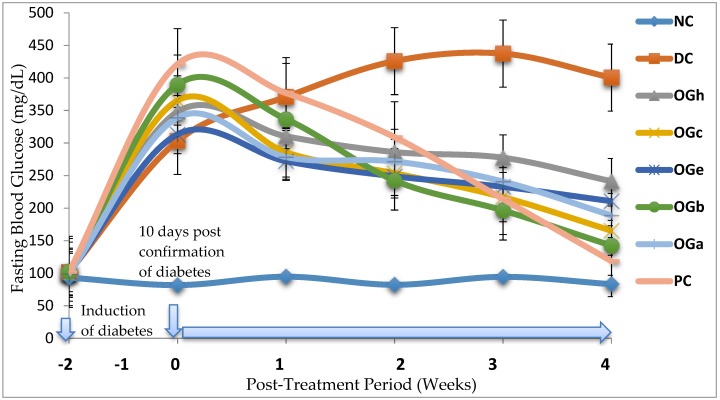
Effect of treatment with *Ocimum gratissimum* leaf fractions (250 mg/kg bw) on fasting blood glucose of diabetic rats. Data are presented as mean of 6 animals per group. NC: Normal control; DC: Diabetic control; PC: Positive control (Metformin 500 mg/kg bw); OGh: *Ocimum gratissimum n*-hexane fraction; OGc: *Ocimum gratissimum* chloroform fraction; OGe: *Ocimum gratissimum* ethyl-acetate fraction; OGb: *Ocimum gratissimum n*-butanol fraction; OGa: *Ocimum gratissimum* aqueous fraction.

In Figure 4 is presented the blood glucose levels (mg/dL) of experimental rats after weekly administration of *Ocimum gratissimum* leaf fraction. A significant (*p* < 0.05) increase in blood glucose was observed after diabetic induction prior to commencement of treatment. After initiation of treatment, gradual decrease in blood glucose was observed among the OGLF treated group and the metformin treated group (PC) when compared with the normal (NC) and the negative (DC) control groups. The blood glucose of the negative control group was significantly (*p* < 0.05) higher than those of the treated groups.

Table 4 shows the percentage decrease in blood glucose as compared weekly in all the groups with respect to their initial blood glucose level recorded after confirmation of diabetes, before commencement of treatment. The results showed a significant difference (*p* < 0.05) in the percentage change in blood glucose level among and within the treated groups.

### 3.6. Effect of Ocimum Gratissimum Leaf Fractions on OGTT and AUC

**Figure 5:**
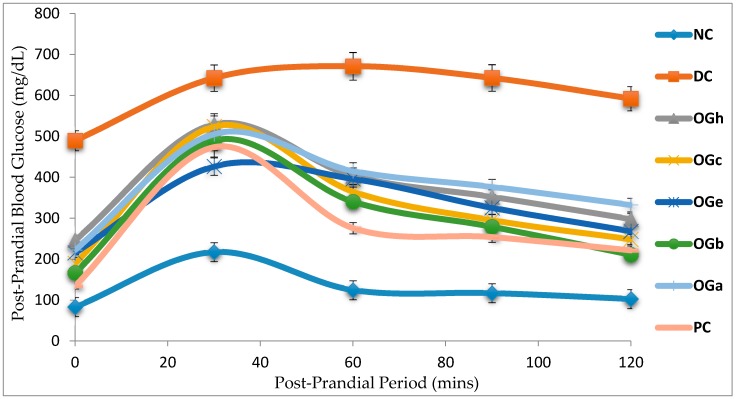
Changes in blood glucose concentration during oral glucose tolerance test in rats treated with *Ocimum gratissimum* leaf fractions (250 mg/kg bw). Data are presented as mean of 6 animals per group. NC: Normal Control; DC: Diabetic control; PC: Positive control (metformin 500 mg/kg bw); OGh: *Ocimum gratissimum n*-hexane fraction; OGc: *Ocimum gratissimum* chloroform fraction; OGe: *Ocimum gratissimum* ethyl-acetate fraction; OGb: *Ocimum gratissimum n*-butanol fraction; OGa: *Ocimum gratissimum* aqueous fraction.

Figure 5 and Table 5 show the blood glucose levels of rats in the normal control, negative control and diabetic groups, pre-treated for 28 days. The OGLF treated groups demonstrated a significant change after oral loading, with 2 g/kg bw glucose solution. The rats in the untreated diabetic group had significant elevation in blood glucose level throughout the total measurement duration (120 min) with respect to the normal control group and OGLF treated groups. More so, the glucose level did not return to the initial values (at 0 min), even at the end of the 2 h period. Treatment of diabetic rats with metformin or OGLF for 28 days resulted in significant reduction in area under the curve (AUC) for glucose when compared to the negative control group. Although the various leaf fractions of OG had hypoglycemic effects, the OG *n*-butanol fraction was observed to have the highest hypoglycaemic effect with 48.1% while the OG aqueous fraction had the least hypoglycaemic activity of 37.0% (Table 5).

### 3.7. Effect of Treatment on Biochemical Parameters

Table 6 shows the levels of serum liver marker enzymes (AST, ALT and ALP) and biomarkers of kidney function (serum urea, uric acid and creatinine) were significantly increased in the negative diabetic control group (DC) as compared to the normal control (NC), positive control (PC) and OGLF treated groups. Oral administration of OGLF to diabetic rats for 4 weeks decreases the levels of serum urea, uric acid, and serum creatinine contents. The *n*-butanol fraction reveals significant (*p* < 0.05) modification.

The effects of the various leaf fractions of OG on serum lipid profile levels in diabetic rats are presented in Table 7. The data obtained showed that serum total cholesterol (Tc), serum triacylglycerol (TG), serum low density lipoprotein cholesterol (LDLc) and total cholesterol ratio high density lipoprotein cholesterol ratio (Tc/HDLc) were significantly raised (*p* < 0.05) in the diabetic negative control group as compared to the normal control group and the OGLF treated groups. But, the high-density lipoprotein (HDLc) levels were significantly lower in the negative control group when compared to the normal control group and the OGLF treated group.

Table 8 shows the HOMA indices for insulin resistance (HOMA-IR) and β-cell function (HOMA-β cell) for the diabetic treated and untreated rats. The results signify that HOMA-IR index was significantly (*p* < 0.05) higher in the negative control group when compared to the normal, positive and OGLF treated groups. There were no significant (*p* > 0.05) differences in the HOMA-IR index between the various leaf fractions of OG. The insulin sensitivity and HOMA-β cell functioning index were significantly lower in the negative control group when compared to the normal group, positive and the OGLF treated group.

The quantitative amount of serum C-peptide and available serum insulin, as well as percentages of HOMA- beta cell function and insulin sensitivity are presented the Table 9. It was observed that the severely hyperglycaemic DC group had 17.3% pancreatic β-cell function (HOMA-β) in the presence of 61.7% of circulatory serum insulin (Table 8). The insulin sensitivity of the 61.7% available circulatory insulin was observed to be 51.5%. Although, the C-peptide levels were significantly (*p* < 0.05) lower in the entire diabetic treated and untreated group when compared with the NC, there was significant (*p* < 0.05) improvement in the beta cell function (>23.5%), insulin sensitivity (>63.2%) and available circulatory serum in insulin both the PC and OGLF treated groups.

### 3.8. Histopathological Examination of the Pancreatic Tissues 

**Figure 6:**
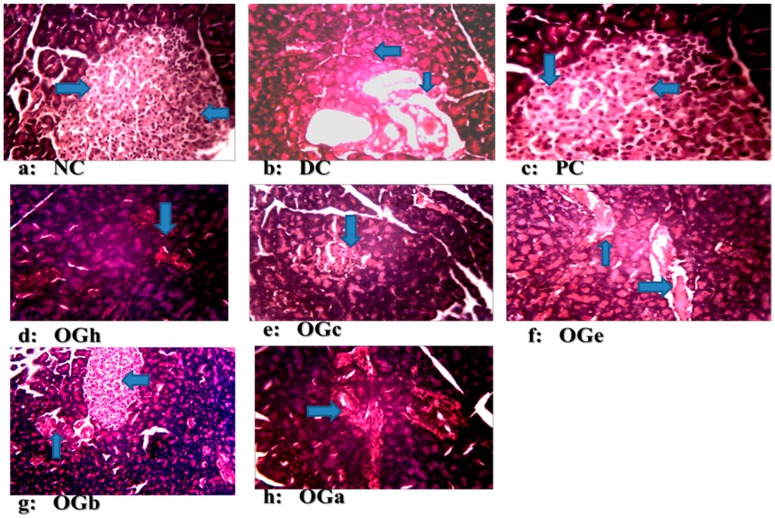
Photomicrographs of pancreatic tissues of diabetic/non-diabetic rats, treated with *Ocimum gratissimum* leaf fractions (250 mg/kg bw) for 28 days (H & E stain 100×). (**a**) NC: Normal control (non-diabetic group): section shows normal histology of pancreas with high number of ß-cells. (**b**) DC: Diabetic control: islets show highly dispersed and morphologically deformed ß-cells. PC: Positive control (Metformin 500 mg/kg bw): it has normal arrangement of islet cells and their ß-cells are likened to that of the NC group. (**c**) OGh: *Ocimum gratissimum n*-hexane fraction; (**d**) OGc: *Ocimum gratissimum* chloroform fraction; (**e**) OGe: *Ocimum gratissimum* ethyl acetate fraction; (**f**) OGb: *Ocimum gratissimum n*-butanol fraction; (**g**) OGa: *Ocimum gratissimum* aqueous fraction. The OGh, OGc, OGe, OGb, OGa groups had better structural architecture compared to the DC group but morphologically distorted ß-cells. The OGh and OGc shows sign of regeneration but cellularity is less pronounced as compared to OGb.

In Figure 6, the photomicrographs of pancreas of the rats in all the experimental groups are presented. The photomicrographs showed pancreatic islets with partial destruction of the β-cells in the negative control group when compared with the normal control and positive control groups with apparently healthy pancreatic β-cells. Treatment with OGLF at a dose of 250 mg/kg bw/day for 4 weeks improves the damaged pancreatic islets cells. A substantial number of healthy pancreatic β-cells were observed in the group treated with *n*-butanol fraction of OG leaf, though the islet cells were appreciably smaller in size compared to the normal control and positive control groups.

## 4. Discussion

The increasing prevalence of T2D threatening the quality of human life demands extensive and qualitative research into development of efficient anti-diabetic agents free of adverse effects. Hence, medicinal plants are constantly being investigated using animal model of the disease with the anticipation of developing a comparatively safe anti-diabetic plant-based product [10,31]. In this study, we reported the anti-diabetic potential of the leaf fractions of *Ocimum gratissimum* in a newly developed unique model of T2D [23]. *Ocimum gratissimum,* although used for the traditional remedy of diabetes mellitus in Nigeria [12,32], the antidiabetic potential of the leaf fractions in T2D model is either not available or scarce in the published literature.

In this study, sequential extraction techniques using different solvents in order of increasing polarity (*n*-hexane, chloroform, ethyl-acetate, *n*-butanol and water) were used to obtain OGLF from the crude methanol extract (since the nature, polarity and solubility of the antidiabetic bioactive constituents in the leaf of OG were not known). In general *n*-hexane is used to extract compounds of low polarity such as fatty acids, waxes, some alkaloids and terpenoids [33]. Chloroform and ethyl-acetate extracts both medium polarities and some polar compounds such as flavonoids, tannins and some terpenoids [33], whereas, *n*-butanol and water extract highly polar compounds like carbohydrates, amino acids and their derivatives [33]. The observed yield of 10.2% methanol crude extract obtained in this study corroborates that reported by Ogundipe, et al. [34] with 10.4%. The presence of pharmacologically active constituents of the various fractions is in agreement with those of previous investigators [35,36].

The oral acute toxicity study of OG leaf extracts at 5000 mg/kg bw did not show mortality or any obvious toxic symptom. This signified that the median lethal dose of (LD_50_) of OG was higher than 5 g/kg bw. This finding was synonymous with that of Egesie, et al. [12] who reported that OG leaf extract elicited no clinical symptoms of toxicity at doses up to 1500 mg/kg/day. Several other investigators have also reported the non-toxic nature of the leaf extract either to the kidney or the liver [12,13,18,34]. Nevertheless, some other investigators had contrary reports including Mohammed, et al. [21] who reported LD_50_ of OG as 1264.9 mg/kg bw, Onaolapo, et al. [20] who reported 912.3 mg/kg bw oral ethanol extract of OG and Okon, et al. who calculated the LD_50_ of OG to be 4242.6 mg/kg bw [17]. However, these differences in the LD_50_ may be due to the age of the plant, geographical location, season and time of harvest, which have been reported to affect the phytochemical compositions of plants [37] and ultimately affect its toxicity.

The effective minimum oral therapeutic dose of OG leaf extract in this study was found to be 250 mg/kg bw. This dose was observed to have reduced hyperglycaemia in diabetic rats by 29.3% at 4 h post oral administration. This finding was consistent with the report of the 25% reduction criteria suggested by Oguanobi et al. [28]. Different doses of OG leaf extracts ranging from 100 to 500 mg/kg bw have been reported by several investigators without clear-cut criteria on how the doses were established [12,22,32,34,38]. The variation in the amount of phytoconstituents could account for the hypoglycemic efficacy of OG leaf extract/fractions at different dosages. The hypoglycaemic potency of OG leaf extract has been attributed to some basic phytochemical constituents present in the plant which include saponins, tannins, flavonoids, phenol, glycosides and steroid glucosides [25]. These phytoconstituents were detected in this study.

Hyperglycaemia in diabetes leads to glycosuria followed by osmotic diuresis (polyuria). This in turn causes water depletion, which activate signals to the thirst centre for increased fluid intake. A vicious series of polyuria events result in polydypsia, the extent of which is subject to the extent of hyperglycaemia [1]. In this study, OGLF significantly (*p* < 0.05) reduced the amount of feed intake, fluid intake and weight loss in the diabetic treated group compared to the DC. Given that water and feed intake are regulated by centres in the hypothalamus, it is possible that OGLF affects these centres. For example, suppression of food intake is a function of the satiety centre in the ventromedial hypothalamus and, water intake is influenced by osmo-regulators in the anterior hypothalamus that sense the osmolality of the body fluids [17]. Consequently, OGLF may be stimulating the satiety centre while inhibiting the thirst centre (osmoreceptors) as well as hunger centre (lateral hypothalamic nuclei) [39].

In this study, polyphagia, polydipsia, polyuria and concomitant reduction in body weight—which are classical symptoms and signs of diabetes [1]—were established in the diabetic negative control group (DC). Increased feed and fluid intake was observed in all the diabetic groups one week after confirmation of diabetes and in addition obvious weight loss was noticed in the DC when compared to the NC and OGLF treated groups. The increase in fluid intake may be the consequence of obligatory renal water loss combined with hyper-osmolarity in diabetes which tends to reduce intracellular water, triggering the osmo-receptor of the thirst centre of the brain and this result in polydipsia which leads to fluid intake [39]. There was a significant reduction in the amount of fluid and quantity of feed intake in the OGLF treated group when compared to the DC group. This could be due to the fact that OGLF improved significantly the classical symptoms of diabetes. Also, the observations suggest that OGLF influences the neuroendocrine regulation of feed intake by the GI system, together with nutrient sensing and peptide secretion by enteroendocrine cells. These findings corroborate previous studies [17,38].

The ability of an animal model to develop obesity, and ultimately diabetes, is one of the most significant criteria for selecting a model [16]. This phenomenon was seen among the experimental rats after six weeks of consumption of margarine fortified died and fructose solution as drinking water prior to treatment with STZ. At four weeks post STZ induction, the relative loss in weight seen among the DC group in spite of the increased feed intake could be due to glucosuria. Glucosuria results in considerable loss of calories. For every gram of glucose excreted together with loss of muscle and adipose tissues, leads to severe weight loss in spite of increase in appetite [40]. Increase muscle degradation could also be the reason for the loss of body weight in DC group. Body weight loss is also reversed by insulin through stimulating protein and lipid synthesis collectively with glycogen storage [41]. The relatively improved body weight gain in the OGLF treated groups as compared to the DC group suggests its antidiabetic efficacy for T2D. In particular, the improved ameliorating effect of OG *n*-butanol leaf fraction may be due to the presence of saponins. Saponins possess insulin-like properties, which stimulate glucose uptake by enhancing Glut4 expression and contributing to storage of glucose as glycogen in adipocytes [42].

The diabetic control rats in this study demonstrated significant increase in AUC of the glucose concentration after oral glucose loading. The data from this study shows that various fractions of OG leaf produced significant (*p* < 0.05) reduction in blood glucose level in T2D rats model. The implication is that OGLF stimulates increased glucose utilization and glucose tolerance through body tissues of the diabetic rats. Among all the OGLF administered, *n*-butanol fraction was observed to have the most significant hypoglycaemic potency in the majority of parameters measured.

Renal diseases are severe complications of diabetes. Serum creatinine, uric acid and urea are common biomarkers for prediction of renal dysfunction, due to the fact that they are elevated considerably in diabetic conditions [43,44]. In this study, high levels of these biomarkers were observed in the DC group as compared to the NC group. This finding is in agreement with that of previous investigators [43,45]. The significant high levels of serum urea and creatinine are signs of reduced capacity of the kidney to sieve these waste products from the blood and expel them in urine. Nevertheless, treatment with OGLF for four weeks was able to significantly (*p* < 0.05) reduce the serum concentration of urea and creatinine in diabetic rats. Among the various fractions administered, it was observed that the OG *n*-butanol fraction was able to significantly reduce the serum levels of urea and creatinine in a manner comparable with the standard drug metformin in the PC group. Data from this study suggest that the *n*-butanol fraction of OG may have improved the capacity of the kidneys to eliminate these waste products from the blood.

Transaminases (AST and ALT) are important and critical enzymes involved in the breakdown of amino acids into α-keto acid, which are routed for complete metabolism through the Krebs cycle and electron transport chain. They are considered specific biomarkers for liver damage [20]. In hepatocyte injury, there is impairment in the liver cell membrane which results in permeability of cytoplasmic enzymes like AST and ALT which escape into the circulatory system and result in an increase in their activities in serum. More so, alteration of membrane bound alkaline phosphatase (ALP) affects membrane permeability and produces derangement in the transport of metabolites [10]. The increased gluconeogenesis and ketogenesis observed in diabetes may be the reason for the high levels of activity of these transaminases [46]. The increase in AST and ALT levels in the serum of diabetic rats reflects impaired liver function. Treatment of diabetic rats with OGLF caused a reduction in the serum activity of the enzymes (AST, ALT, ALP). This observation is in harmony with reports from other investigators that elevated activities of serum transferases are common signs of liver diseases observed more often in diabetic conditions [10,12,45]. The decrease in the enzyme levels in PC group shows that metformin prevented liver damage. Among the various OGLF tested, the *n*-butanol fraction of OG was the most active in reducing the activities of the liver enzymes. The return of the biomarker enzymes to near normal serum values following OGLF treatment may be due to prevention of intracellular enzyme leakage resulting from cell membrane stability or cellular regeneration. This suggests that the leaf fractions of OG possess hepatoprotective potential.

The observed high levels of total cholesterol (Tc), triglyceride (TG) and low-density lipoprotein cholesterol (LDLc) of diabetic rats are in accord with previous reports documented on elevated levels of TG, LDL and Tc in diabetic subject [10,38]. Hyperlipidaemia is a complication of diabetes and has been reported to be due to excess mobilization of fat from the adipose tissues due to under-utilization of glucose [47] or inhibition of the hormone sensitive lipase by insulin [48]. In this study, the observed hypercholesterolaemia may be attributed to the increased dietary cholesterol absorption from the small intestine following the intake of FDF in a diabetic condition [23]. However, on treatment of the diabetic groups with OGLF, there were significant reductions in TG, LDL and Tc levels, specifically in the *n*-butanol fraction group. This observation is in consonance with the findings that most hypoglycaemic plants have potentials of ameliorating diabetic lipid metabolism anomalies [38]. It has been reported previously that alkaloids, flavonoids, saponin and cardiac glycosides possess a hypolipidaemic effect in animals [49]. These phytochemicals were present in the OGLF. In addition, saponins may decrease cholesterol by binding with cholesterol in the intestinal lumen, thereby inhibiting its absorption and/or by binding with bile acids and causing a decline in the enterohepatic circulation of bile acids and increase in its faecal excretion [50]. The increased bile acid excretion is equalized by enhanced bile acid production from cholesterol in the liver and consequent lowering of the plasma cholesterol.

The photomicrograph in this study reveals normal pancreatic islets β-cells in the normal control group, with the diabetic negative control group reflecting evidence of deformation and not complete destruction associated with Type-1 diabetes. This includes cytoplasmic changes in the cells of the islets, particularly within the core portion of the islets where there was architectural deformation of β-cells. The normal and positive control groups had large islet cells with high number of β-cells while the DC had morphologically deformed islet cells with disperse β-cells. The OGLF treated groups had higher (but morphologically smaller) number of β-cells compared to DC, but fewer numbers compared to NC. The OGLF treated groups showed regeneration of the β-cells of the pancreas, and this observation was evidently supported by the significant reduction in hyperglycaemia. These changes were observed consistently in all the animals in the groups. However, *n*-butanol fraction of the OG leaf manifested the greatest pancreas regenerative tendency compared to the other groups. This study further confirms the potency of OGLF.

The experimental model of T2D used in the present investigation is a typical example of the unhealthy lifestyle seen globally in human T2D subjects, particularly of western origin. In this case, fructose solution (20%) in drinking water is consumed together with a high fat diet (margarine fortified diet feed) to induce insulin resistance. This rat model is a unique non-genetic Type-2 diabetic model as it presented considerable increase insulin resistance and decrease insulin sensitivity without absolute serum insulin deficiency. The 61.7% available serum insulin observed in this study among the untreated diabetic rats at the end of the experiment suggests that insulin secretory defect in T2D is due to impairment of insulin action [51,52]. This percentage of available insulin observed is in agreement with previous models of T2D reported by other investigators [23,52,53,54,55]. Contrary to the T2D model used in this study, almost absolute insulin deficiency is associated with T1D [56,57]. For instance, in an earlier study by Dnansh et al. [58], available serum insulin in the T1D model was 28.2%, 21.7% and 19.8% after 15, 30 and 45 days respectively of STZ induction. A more recent study demonstrated that the available serum insulin in STZ induced T1D model was less than 46.8% at the point of confirmation of diabetes [23]. Nevertheless, Wilson and Islam [59] reported 53.7% serum insulin in fructose-STZ induced T2D model whereas Suman et al. [60] reported 45.5% for T2D.

It has been established that as T2D progresses, there is a gradual decline in pancreatic beta-cell function which ultimately results in β-cell failure [16,54,56], the consequence of which is a continuous decrease in the amount of available serum insulin. Previous report suggested that the available circulatory serum insulin greater than 85.8% should be used for selecting T2D model for pharmacological screening [23]. Interestingly, although only 17.3% of pancreatic β-cell function (HOMA-β) were available in the untreated diabetic group, a high serum insulin concentration of 61.7% was noticed at 38-day post STZ induction. This observation could be due to the compensatory tendency of the remaining pancreatic β-cells to secrete more insulin.

## 5. Conclusions

This study showed that the daily administration of *n*-hexane, chloroform, ethyl acetate, *n*-butanol and aqueous fractions of *Ocimum gratissimum* leaf resulted in reduction in blood glucose levels in the new animal model of T2D. The beneficial effects of the OG leaf fractions could be attributed to improved insulin sensitivity and beta-cell function as a result of alkaloids, flavonoids, saponins and/or tannins present in the fractions. These phytochemicals are known to possess antidiabetic activities [25] and at least one or more of them were present in the fraction. The data from this study gives credibility to existing reports that OG is valuable in the ethno-therapeutic management of diabetes mellitus [12,19,20,22,32]. Apart from the fact that all five fractions were confirmed to be effective, the *n*-butanol fraction of OG was revealed to be the most effective, since it had the satisfactory ability to revert diabetic alterations to near normal. Even though the intervention period in the study was rather short at just four weeks, it shows promise of the effects of OG *n*-butanol fraction being improved in a subsequent longer-term study. Work is on the way in our laboratory to isolate and identify the bioactive components present in the *n*-butanol fraction of *Ocimum gratissimum.*

## Figures and Tables

**Table 1:** Percentage recovery yield of *Ocimum gratissimum* leaf extract and fractions.

Extract/Fraction	Colour	Weight of Yield (g)	Recovery Yield (%)
Crude extract	Blackish green	126.8 ± 2.3	10.2
*n*-Hexane fraction	Black green	13.2 ± 0.7	10.4
Chloroform fraction	Dark green	6.4 ± 1.1	5.1
Ethyl acetate fraction	Brownish	17.5 ± 0.9	13.8
*n*-Butanol fraction	Light brown	2.8 ± 1.8	2.2
Aqueous fraction	Brown	42.7 ± 1.4	33.7

Values are mean ± SD of duplicate determinations.

**Table 2:** Some phytochemicals detected in *Ocimum gratissimum* leaf fractions.

S/N	Phytochemicals	Leaf Fraction				
		*n*-Hexane	Chloroform	Ethyl-Acetate	*n*-Butanol	Aqueous
1	Alkaloids	+	+	+	++	−
2	Carbohydrate	+	+	+	++	+
3	Anthraquinones	−	−	−	−	−
4	Glycoside	+	+	++	+	+
5	Flavonoids	−	++	+	++	+
6	Polyphenols	+	+	+	++	−
7	Saponins	−	−	−	++	+
8	Steroids	+	+	+	−	−
9	Tannins	−	−	−	+	+
10	Terpenoids	−	+	−	+	−

(+++): Highly present; (++): Moderately present; (+): Present; (−): Absent/Not detected.

**Table 3:** Determination of effective minimum oral therapeutic dose of *Ocimum gratissimum* methanol leaf extract in rat model of type-2 diabetes.

OG Extract Dose (mg/kg bw)	Blood Glucose Level (mg/dL)			
	0 h	1 h	2 h	4 h
G_100_	261.4 ± 12.4	247.8 ± 14.3 (5.1)	241.7 ± 11. 8 (6.3)	237.2 ± 9.1 (9.3)
G_150_	284.1 ± 15.8	270.6 ± 9.4 (4.8)	259.5 ± 8.5 (8.7)	249.3 ± 3.6 (12.3)
G_200_	273.7 ± 20.6	243.4 ± 14.5 (11.1)	237.2 ± 14.3 (13.3)	216.6 ± 17.1 (20.9)
G_250_	291.3 ± 14.9	260.5 ± 22.2 (10.6)	241.4 ± 15.8 (17.1)	206.1 ± 20.5 (29.3)
DC	286.9 ± 15.5	282.3 ± 15.1 (1.6)	277.7 ± 12.2 (3.2)	274.2 ± 12.3 (4.4)

Figure in brackets is percentage reduction in blood glucose as compared to Values obtained at 0 h. Data are presented as mean ± SD of 3 animals per group. DC: Diabetic control group without treatment. OG: *Ocimum gratissimum*.

**Table 4:** Percentage decrease in fasting blood glucose of diabetic rats, after treatment with *Ocimum gratissimum* leaf fractions (250 mg/kg bw).

Groups	FBS (mg/dL) Day 0	Percentage Decrease after Treatment (%)			
		Day 7	Day 14	Day 21	Day 28
NC	83.7 ± 5.9 ^a^	2.4 ± 6.1 ^b,1^	1.6 ± 7.0 ^b,1^	−0.9 ± 5.6 ^b,1^	0.6 ± 5.6 ^b,1^
DC	303.3 ± 15.7 ^b^	−22.3 ± 7.0 ^a,3^	−40.6 ± 8.0 ^a,1,2^	−44.6 ± 13.0 ^a,1,2^	−50.5 ± 11.5 ^a,1^
OGh	347.9 ± 7.9 ^c,d^	10.8 ± 4.4 ^b,c,1^	17.8 ± 3.9 ^c, 1,2^	20.3 ± 3.8 ^c,2^	30.6 ± 5.0 ^c,3^
OGc	365.4 ± 14.6 ^d^	21.8 ± 3.0 ^d,1^	30.6 ± 7.5 ^d,e,1,2^	40.6 ± 6.4 ^d,2^	54.7 ± 6.6 ^e,3^
OGe	313.2 ± 21.1 ^b^	12.8 ± 9.4 ^c,1^	20.4 ± 9.9 ^c,d,1,2^	25.6 ± 8.9 ^c,1,2^	32.6 ±5.10 ^c,2^
OGb	389.2 ± 10.2 ^e^	13.6 ± 5.3 ^c,d,1^	37.9 ± 7.9 ^e,2^	49.7 ± 7.3 ^d,2^	63.5 ± 4.8 ^f,3^
OGa	339.0 ± 8.0 ^c^	17.9 ± 4.0 ^c,d,1^	19.9 ± 4.0 ^c,1^	29.0 ± 3.6 ^c,2^	44.4 ± 10.9 ^d,3^
PC	421.2 ± 38.2 ^f^	10.1 ± 11.5 ^b,c,1^	26.7 ± 14.1 ^d,1,2^	49.1 ± 7. 8 ^e,2^	71.7 ± 2.2 ^f,3^

Data are presented as mean ± SD of 6 animals per group. Values with different superscript down the column and different alphabet across the column indicate significant difference (*p* < 0.05). NC: Normal control; DC: Diabetic control; PC: Positive control (Metformin 500 mg/kg bw); OGh: *Ocimum gratissimum n*-hexane fraction; OGc: *Ocimum gratissimum* chloroform fraction; OGe: *Ocimum gratissimum* ethyl-acetate fraction; OGb: *Ocimum gratissimum n*-butanol fraction; OGa: *Ocimum gratissimum* aqueous fraction. Negative sign (−) before a figure signifies increase in fasting blood glucose.

**Table 5:** Total area under the curve induced after oral glucose loading (2.0 g/kg bw) in diabetic rats treated with *Ocimum gratissimum* leaf fractions (250 mg/kg/day) for 28 days.

Groups	Total AUC (mg/dL min)	Reduction (%)
NC	16,538 ± 429.2	–
DC	74,922 ± 1045.2	0.0
OGh	46,697 ± 423.4	37.7
OGc	42,095 ± 348.9	43.8
OGe	40,606 ± 577.9	45.8
OGb	38,921 ± 174.9	48.1
OGa	47,241 ± 933.5	37.0
PC	47,418 ± 113.5	36.7

Values are mean ± SD of 6 rats. NC: Normal control; DC: Diabetic control; PC: Positive control (Metformin 500 mg/kg bw) OGh: *n*-Hexane fraction; OGc: Chloroform fraction; OGe: Ethyl acetate Fraction; OGb: *n*-Butanol; Aqueous OGa.

**Table 6:** Liver and kidney function biomarkers in diabetic rats treated with *Ocimum gratissimum* leaf fractions (250 mg/kg/day) for 28 days.

Groups	AST (U/I)	ALT (U/I)	ALP (U/I)	Urea (mg/dL)	Creatinine (mg/dL)	Uric Acid (mg/dL)
NC	27.7 ± 2.9 ^a,b^	23.4 ± 4.9 ^a^	32.7 ± 7.4 ^a^	25.8 ± 2.4 ^a^	0.9 ± 0.1 ^a^	2.5 ± 0.3 ^a^
DC	69.7 ± 6.4 ^d^	62.4 ± 8.4 ^f^	97.8 ± 13.7 ^e^	40.7 ± 2.5 ^c^	2.2 ± 0.2 ^e^	4.8 ± 0.7 ^c^
OGh	41.4 ± 6.0 ^c^	35.9 ± 5.6 ^c,d^	57.13 ±8.6 ^b,c^	30.2 ± 3.1 ^b^	1.5 ± 0.4 ^c,d^	3.2 ± 0.6 ^b^
OGc	37.5 ± 2.9 ^c^	42.7 ± 2.2 ^d,e^	70.3 ± 12.6 ^d^	31.6 ± 2.5 ^b^	1.4 ± 0.2 ^b,c^	2.9 ± 0.3 ^a,b^
OGe	30.5 ± 4.1 ^a,b^	46.7 ± 5.7 ^e^	64.7 ± 7.8 ^c,d^	30.1 ± 2.3 ^b^	1.7 ± 0.1 ^d^	3.4 ± 0.3 ^b^
OGb	26.1 ± 2.9 ^a^	25.4 ± 5.3 ^a,b^	33.2 ± 7.4 ^a^	29.6 ± 2.4 ^a,b^	1.1 ± 0.3 ^a,b^	2.8 ± 0.5 ^a,b^
OGa	42.7 ± 5.1 ^c^	32.0 ± 6.1 ^b,c^	51.2 ± 6.0 ^b^	32.9 ± 2.5 ^b^	1.7 ± 0.2 ^c,d^	3.2 ± 0.4 ^b^
PC	32.0 ± 1.4 ^d^	29.8 ± 10.0 ^a,b,c^	49.5 ± 14.9 ^b^	29.7 ± 2.2 ^a,b^	1.4 ± 0.2 ^b,c^	3.0 ± 0.3 ^a,b^

Data are presented as mean ± SD of 6 animals per group. Values with different superscript down the column indicate significant difference (*p* < 0.05). AST: Aspartate transaminase; ALT: Alanine transaminase; ALP: Alkaline phosphatase; NC: Normal control; DC: Diabetic control; PC: Positive control (Metfomin 500 mg/kg bw); OGh: *Ocimum gratissimum n*-hexane fraction; OGc: *Ocimum gratissimum* chloroform fraction; OGe: *Ocimum gratissimum* ethyl-acetate fraction; OGb: *Ocimum gratissimum n*-butanol fraction; OGa: *Ocimum gratissimum* aqueous fraction.

**Table 7:** Effects of treatment with *Ocimum gratissimum* leaf fractions (250 mg/kg bw) for 28 days on serum lipid profile of diabetic rats.

Groups	TC (mg/dL)	HDLC (mg/dL)	TG (mg/dL)	LDLC (mg/dL)	TC/HDL (mg/dL)
NC	78.0 ± 7.6 ^a^	58.6 ± 5.8 ^e^	73.6 ± 8.0 ^a^	4.7 ± 9.2 ^a^	1.3 ± 0.2 ^a^
DC	176.7 ± 11.8 ^d^	17.8 ± 2.6 ^a^	448.7 ± 11.4 ^e^	69.1 ± 10.3 ^c,d^	10.1 ± 1.3 ^f^
OGh	109.5 ± 10.3 ^b^	35.1 ± 3.3 ^c^	119.2 ± 7.4 ^d^	50.6 ± 13.2 ^b,c^	3.2 ± 0.5 ^b,c^
OGc	117.1 ± 10.7 ^b^	32.3 ± 4.7 ^b,c^	99.2 ± 5.6 ^c^	65.0 ± 9.7 ^c^	3.7 ±0.5 ^c,d^
OGe	111.2 ± 7.7 ^b^	27.5 ± 4.9 ^b^	115.1 ± 5.7 ^d^	60.7 ± 8.9 ^b,c^	4.1 ± 0.7 ^d,e^
OGb	102.6 ± 24.6 ^b^	41.4 ± 6.6 ^d^	87.0 ± 6.8 ^b^	43.8 ± 24.3 ^b,c^	2.5 ± 0.6 ^b^
OGa	117.4 ± 18.9 ^b^	28.2 ± 5.3 ^b^	101.8 ± 6.8 ^c^	68.8 ±18.1 ^c,d^	4.3 ± 0.8 ^d,e^
PC	137.5 ± 12.8 ^c^	29.6±5.9 ^b,c^	105.2 ± 6.3 ^c^	86.9 ± 17.7 ^d^	4.8 ± 1.2 ^e^

Data are presented as mean ± SD of 6 animals per group. Values with different superscript down the column indicate significant difference (*p* < 0.05). TC: Serum Total cholesterol; HDLC: Serum High density lipoprotein cholesterol; LDLC: Serum Low density lipoprotein cholesterol; TG: Serum Triglyceride; NC: Normal control; DC: Diabetic control; PC: Positive control (Metformin 500 mg/kg bw); OGh: *Ocimum gratissimum n*-hexane fraction; OGc: *Ocimum gratissimum* chloroform fraction; OGe: *Ocimum gratissimum* ethyl-acetate fraction; OGb: *Ocimum gratissimum n*-butanol fraction; OGa: *Ocimum gratissimum* aqueous fraction.

**Table 8:** Quantification of insulin sensitivity, resistance and beta cell function in diabetic rats treated with *Ocimum gratissimum* leaf fractions (250 mg/kg bw) for 28 days.

Groups	FBS(mg/dL)	HOMA-IR	HOMA-β	Insulin Sensitivity
NC	83.1 ± 6.4 ^a^	2.7 ± 0.4 ^a^	52.3 ± 5.6 ^e^	1.4 ± 0.1 ^e^
DC	489.6 ± 53.8 ^f^	12.4 ± 2.8 ^d^	4.0 ± 0.9 ^a^	0.7 ± 0.1 ^a^
OGh	246.4 ± 19.1 ^e^	6.5 ± 0.6 ^c^	12.3 ± 3.0 ^b^	0.9 ± 0.0 ^b^
OGc	189.8 ± 17.8 ^c,d^	5.0 ± 0.5 ^b,c^	16.9 ± 3.1 ^b^	1.0 ± 0.1 ^b,c^
OGe	215.6 ± 20.2 ^d,e^	5.8 ± 1.0 ^c^	14.6 ± 2.3 ^b^	0.9 ± 0.1 ^b,c^
OGb	166.3 ± 16.9 ^b,c^	4.8 ± 1.0 ^b,c^	21.6 ± 3.2 ^c^	1.0 ± 0.1 ^c^
OGa	222.7 ± 55.4 _d,e_	6.2 ± 2.0 ^c^	15.3 ± 5.4 ^b^	0.9 ± 0.2 ^b,c^
PC	134.1 ± 12.9 ^b^	3.86 ± 0.5 ^a,b^	28.16 ± 5.6 ^d^	1.1 ± 0.1 ^d^

Data are presented as mean ± SD of 6 animals per group. Values with different superscript down the column indicate significant difference (*p* < 0.05). Conversion factor: insulin (1 U/L = 7.174 pmol/L). NC: Normal control; DC: Diabetic control; PC: Positive control (Metformin 500 mg/kg bw); OGh: *Ocimum gratissimum n*-hexane fraction; OGc: *Ocimum gratissimum* chloroform fraction; OGe: *Ocimum gratissimum* ethyl-acetate fraction; OGb: *Ocimum gratissimum n*-butanol fraction; OGa: *Ocimum gratissimum* aqueous fraction.

**Table 9:** Serum C-peptide and circulating serum insulin concentrations and percentages of HOMA-β cell function and insulin sensitivity in diabetic rats treated with *Ocimum gratissimum* leaf fraction (250 mg/kg bw) for 28 days.

Groups	Percentage β-Cell Function (%)	Circulating Serum C-Peptide (nmol/L)	Percentage Insulin Sensitivity (%)	Available Circulating Serum Insulin (mU/L)	Percentage Improvement in Insulin Sensitivity (%)	Percentage of Circulating Serum Insulin (%)
NC	100.0	0.8 ± 0.1 ^b^	100.0	15.5 ± 1.9 ^c^	-	100.0
DC	17.3	0.5 ± 0.1 ^a^	51.5	9.6 ± 1.3 ^a^	0.0	61.7
OGh	23.5	0.5 ± 0.2 ^a^	63.2	10.7 ± 1.4 ^a,b^	22.9	69.0
OGc	33.2	0.5 ± 0.1 ^a^	69.9	10.6 ± 1.0 ^a,b^	35.7	68.7
OGe	27.8	0.5 ± 0.0 ^a^	67.7	10.8 ± 1.3 ^a,b^	31.4	69.6
OGb	41.3	0.6 ± 0.1 ^a^	71.3	11.6 ± 1.6 ^b^	38.6	74.8
OGa	29.3	0.5 ± 0.2 ^a^	65.4	11.0 ± 1.5 ^a,b^	27.1	71.2
PC	53.8	0.7 ± 0.3 ^a^	80.2	11.7 ± 1.5 ^b^	55.7	75.5

Data are presented as percentages or mean ± SD of 6 animals per group. Values with different superscript down the column indicate significant difference (*p* < 0.05). Conversion factor: insulin (1 U/L = 7.174 mol/L). NC: Normal control; DC: Diabetic control; PC: Positive control (Metformin 500 mg/kg bw); OGh: *Ocimum gratissimum n*-hexane fraction; OGc: *Ocimum gratissimum* chloroform fraction; OGe: *Ocimum gratissimum* ethyl-acetate fraction; OGb: *Ocimum gratissimum n*-butanol fraction; OGa: *Ocimum gratissimum* aqueous fraction.
